# Multi-color and Multi-Material 3D Printing of Knee Joint models

**DOI:** 10.1186/s41205-021-00100-0

**Published:** 2021-04-29

**Authors:** Oliver Grimaldo Ruiz, Yasin Dhaher

**Affiliations:** 1 Department of Structural, Geotechnical and Building Engineering (DISEG), Politecnico di Torino, 10129 Turin, Italy; 2 Physical Medicine and Rehabilitation, Orthopaedic Surgery, UT Southwestern Medical Center, 5323 Harry Hines Blvd, Dallas, TX 75390 USA

**Keywords:** Three-dimensional printing, Knee Joint, Anatomical models, Anterior cruciate ligament reconstruction, Total knee arthroplasty

## Abstract

**Objective:**

This study reports on a new method for the development of multi-color and multi-material realistic Knee Joint anatomical models with unique features. In particular, the design of a fibers matrix structure that mimics the soft tissue anatomy.

**Methods:**

Various Computer-Aided Design (CAD) systems and the PolyJet 3D printing were used in the fabrication of three anatomical models wherein fibers matrix structure is mimicked: (i) Anterior cruciate ligament reconstruction (ACL-R) model used in the previous study. (ii) ACL-R model, incorporating orientations, directions, locations, and dimensions of the tunnels, as well as a custom-made surgical guide (SG) for avoiding graft tunnel length mismatch. (iii) Total knee arthroplasty (TKA) model, including custom-made implants. Before models 3D printing, uni-axial tensile tests were conducted to obtain the mechanical behaviors for individual No. 1 (A60-A50), No. 2 (A50-A50), No. 3 (A50-A40), and No. 4 (A70-A60) soft tissue-mimicking polymers. Each material combination represents different shore-hardness values between fiber and matrix respectively.

**Results:**

We correlated the pattern of stress-strain curves in the elastic region, stiffness, and elastic modulus of proposed combinations with published literature. Accordingly, material combinations No. 1 and No. 4 with elastic modules of 0.76-1.82 MPa were chosen for the soft tissues 3D printing. Finally, 3D printing Knee Joint models were tested manually simulating 50 flexo-extension cycles without presenting ruptures.

**Conclusion:**

The proposed anatomical models offer a diverse range of applications. These may be considered as an alternative to replacing cadaver specimens for medical training, pre-operative planning, research and education purposes, and predictive models validation. The soft tissue anatomy-mimicking materials are strong enough to withstand the stretching during the flexo-extension. The methodology reported for the design of the fiber-matrix structure might be considered as a start to develop new patterns and typologies that may mimic soft tissues.

## Background

Three-dimensional (3D) printing is an emerging technology that is getting substantial interest over the past years in several key areas such as the automotive, aerospace, and especially medicine. The impact of 3D printing in the medical field has acquired considerable relevance in the scientific and academic communities owing to its growth in both facility adoption and a wide range of applications [1–3]. However, the 3D printing role in medicine is not recent, this has been reported since the early 1990s and in recent years, there has been a considerable rise in the number of emerging trends in the field, demonstrated by the growing body of literature featuring clinical work and medical research [4].

The Medical 3D printing advancement is the result of the convergence of multiple factors driven by lower-cost, access, and evolution of 3D printing software and hardware, faster 3D printing hardware with multicolor and multi-material capabilities, availability of a wider range of new 3D printing materials, interoperability standards, and cloud-based workflow management tools, further support of industry stakeholders as well as increased commitment from medical societies and regulators [3, 5]. Current research applications are classified into the following five main areas of focus: (i) Anatomical models derived from medical images, (ii) Custom-made prosthetics and implants, (iii) Local bioactive and biodegradable scaffolds, (iv) Pharmaceutical research platforms, and (v) Research on directly printing tissues and organs with complete life functions. Although, such applications remain far from widespread in clinical use due to several technical and scientific issues that are currently under study [6]. In particular, 3D printing anatomical models are becoming increasingly popular and accessible owing to their multiple applications in pre-operative planning, intraoperative navigation, surgical treatments analysis, design and shape of medical devices such as fixation plates and catheters before intervention, to build patient-specific surgical instruments, and for medical training, education and research purposes [4, 7].

In the Knee Joint, most investigations conducted are focused predominantly on total knee arthroplasty (TKA) and their importance in the development of patient-specific instrumentation (PSI), and custom-made implants [8–10]. Nevertheless, anterior cruciate ligament (ACL) tear remains the most frequently performed intra-articular surgery in orthopedic trauma [11, 12]. Despite its prevalence and impact, the number of publications about the 3D printing applications in ACL reconstruction (ACL-R) has been relatively unexplored. The design of patient-specific ACL femoral tunnel guide [13] and a method of accurate bone tunnel placement for ACL-R [14] are the topics most highlighted in publications. This statement does not consider other important studies and contributions about surgical and numerical simulations based on 3D computer-generated anatomical models.

The ACL-R aims to restore physiological joint biomechanics in symptomatic knee, critical factors divided into three categories: (i) Post-operative traumatic injuries, (ii) Lack of graft incorporation and (ii) Surgical technical errors (graft-related, femoral and tibial tunnel malposition, and failure of fixation) are the main problems associated with unsatisfactory medium to long-term clinical outcomes [15, 16]. Indeed, surgical technique-related errors are the most common cause of relapsing instability after ACL-R, accounting for an average of 86% of all cases of ACL failure [17]. Besides, several studies have reported the rise of the development of premature degenerative diseases such as osteoarthritis (OA) because of a failed ACL-R. Thus far, there are no available interventions-treatments to restore degraded structures or decelerate disease development. During OA progression. In an advanced stage, TKA is considered as the most suggested surgical procedure to restore mechanical axes, correct alignment, and soft-tissue balance [18].

In orthopedics, 3D printing anatomical models provide essential information such as initial condition, sizes, directions, positions, and angulations of the bones and surrounding soft tissues. Indeed, researchers and surgeons use this preliminary knowledge for studying complex cases, teaching students and patients, rehearsing the procedures in risk-free settings, pre-procedure designing of grafts, surgical instruments and implants, and as a diagnostic tool [19, 20].

This study reports on a new method for the development of multi-color and multi-material realistic Knee Joint anatomical models with unique features. In particular, the design of a fibers matrix that mimics the soft tissue anatomy. We manufactured and tested three Knee Joint models wherein fibers matrix structure is incorporated. Our models integrated key surgical outcomes of the ACL-R computational framework using a bone-patellar tendon-bone (BPTB) auto-graft and a custom-made SG for avoiding graft tunnel length mismatch. Furthermore, we designed a model after the TKA procedure considering a custom-made cruciate sacrificing (CS) implant with symmetric tibial bearing design and assembling the implants to the healthy Knee Joint model.

## Materials and Methods

### Multi-color and multi-material three-dimensional printing

The present study was developed in the Shirley Ryan Abilitylab research hospital which has a Stratasys J750 (Stratasys, Eden Prairie, MN) multi-color and multi-material 3D printer. This system was used in the fabrication of all soft tissue-mimicking polymers (specimens) and Knee joint models. The printer is operated with GrabCAD Print software to load the final assemblies files, assign printing material, and set up print mode to each sample.

The Stratasys J750 uses 3D PolyJet technology, the printer consists of four photopolymer heads depositing from eight reservoirs, which are linked to material print cartridges; two UV light sources cure the photosensitive resin as one roller runs over the samples layer-by-layer. This system has a large manufacturing tray; the maximum build size of a prototype is 490 x 390 x 200 mm. The 3D printing process enables simultaneously mixing of up to six different materials and adjust the material hardness according to the Shore A scale. Other capabilities to include accuracy of up to 0.2 mm and smaller layer thickness (LT) of 0.014 mm. This system has three print modes in line with the desired surface finish, production time, and the number of materials after incorporated [1]. High Quality six different materials (0.014mm LT), [2] High Mix six different materials (0.027mm LT) and [3] High Speed three different materials (0.027mm LT) [21, 22].

The available 3D printing materials belong to two families: the Digital model, and Model resins. The first one corresponds to engineering plastic acrylonitrile butadiene styrene (ABS) in their versions Digital ABS Plus - Digital ABS2 Plus (main material used in Fused Deposition Modeling (FDM) technology). The second one includes primary materials options: Vero family (rigid opaque materials), RGD525 (high-temperature resistant materials), DurusWhite (simulated polypropylene materials), Tango-Agilus30 (rubber-like materials), and VeroClear - RGD720 (transparent materials) [23]. One type of material, Agilus30 (FLX935) with different shore-hardness values between fiber and matrix was chosen to fabricate all soft tissue-mimicking polymers. After the selection and matching process of the tested specimens, three types of materials for Knee Joint models 3D printing were chosen, Agilus30 (FLX935), Tango (FLX930), and Digital ABS (RGD5130).

### Image data management

**Fig. 1:**
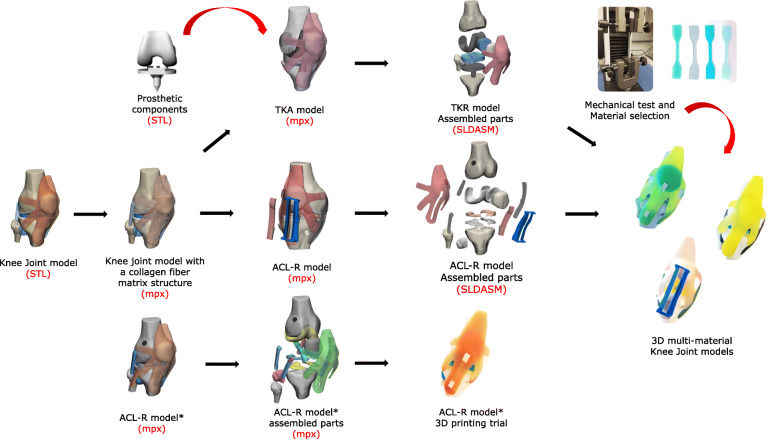
Schematic illustration of the formats used in this study. From the healthy Knee Joint model, the fiber matrix that mimics the hierarchical structure of soft tissues is designed. Thereafter Three paths are shown: (i) ACL-R model* used in the previous study without fiber matrix. (ii) ACL-R model integrating surgical outcomes of the ACL-R computational framework and a custom-made SG for avoiding graft tunnel length mismatch. (iii) TKA model, including custom-made implants

Three 3D computer-generated anatomical models were proposed from the healthy Knee joint model: (i) ACL-R model* used in the previous study, (ii) ACL-R model, and (iii) TKA model. They were based on standard triangle language (STL) files corresponding to the previously developed right male Knee Joint [24]. The initial model incorporates patello-femoral (PF) and tibio-femoral (TF) joints. The following anatomical structures were included: femur, tibia, patella, fibula, major ligaments, articular cartilage, menisci, retinacula, and patella and quadriceps tendons (PT-QT). The 3D computer-generated anatomical models were developed in Materialise 3-Matic (Materialise NV, BE), a design and meshing software for anatomical data. They were exported in STL format to the CAD software SolidWorks, (Dassault Systèmes, France) where they were converted to SolidWorks part file (SLDPRT) format, before being assembled through the same application. The SolidWorks final assembly format (SLDASM) was compatible with the Stratasys GrabCAD print software of the Stratasys J750 multi-material printer where print mode, orientation, and materials were set. The workflow illustrated in Fig. 1. shows the different file formats used in this study. The printing materials selection and matching with the real Knee joint structures were based on the results of the mechanical tensile test. The format extensions and file names are associated with a software application, which opens, manages, and saves these types of files used in this study. These are shown in Table 1.

**Table 1:** 3D File formats used in the current study. Extensions, file names, and software applications

Format extension	Filename	Software application
(.3dm)	3D Object	Rhinoceros 3D
(.mxp)	Project	Materialise 3-Matic
(.SLDPRT)	3D Object	SolidWorks
(.SLDASM)	Assembly	SolidWorks
(.print)	Print project	GrabCAD Print
(.STL)	Standard triangle language	Global CAD software

### Design of the fibers matrix structure that mimics the soft tissue anatomy

Knee Joint specialized connective soft tissues play a crucial role, providing strength, transmitting mechanical loads, and contributing to passive support and stability. Indeed, all these functions are made possible by their hierarchical organization. In particular, tendons and ligaments share many similar features. They are load-bearing structures, their high tensile strength ∼50-150 MPa and their elastic modulus ~1-2 GPa provide all the functional requirements associated with locomotor movement. As expected, both easily bend and change shape to accommodate changes in joint position and skeletal orientation [25].

Highly paralleled collagen fibrous units characterize tendons and ligaments. Accordingly, it may be argued that these tissues are analogous to engineering fiber composites where fibers are laid down in parallel for directional reinforcement [26]. The matrix of collagen fibrils aligned (approximate diameter Ø collagen fibril 1.5 nm) is organized into long cross-striated fibrils that are arranged in bundles to form fibers (approximate diameter Ø fiber 50-500 nm). Fibers are further grouped in arrays called fascicles (Ø fascicle 50-300 μm), and these arrays together form the ligament (Ø ligament fiber 0.1-0.5 mm) [27]. In the Knee Joint, the hierarchical structure of connective tissues described determines the mechanical behavior. Therefore, the knowledge of a structure's mechanical properties is essential to elucidate behavior and function, as well as for selecting appropriate materials used in surgical reconstructive procedures.

To mimic the collagen fibers matrix structure, the STL files of the initial Knee Joint model were exported to Materialise 3-Matic software. A frequent problem in 3D objects management is the relative position. In general, there is no match between the global reference system (GRS) of the different applications. The Materialise 3-Matic software integrates orientation tools (translate & rotate) for precise positioning of anatomical components according to anatomical references. The first step in fiber design was to establish the orientation of the Knee Joint about the anatomical and GRS planes of the application.

**Fig. 2:**
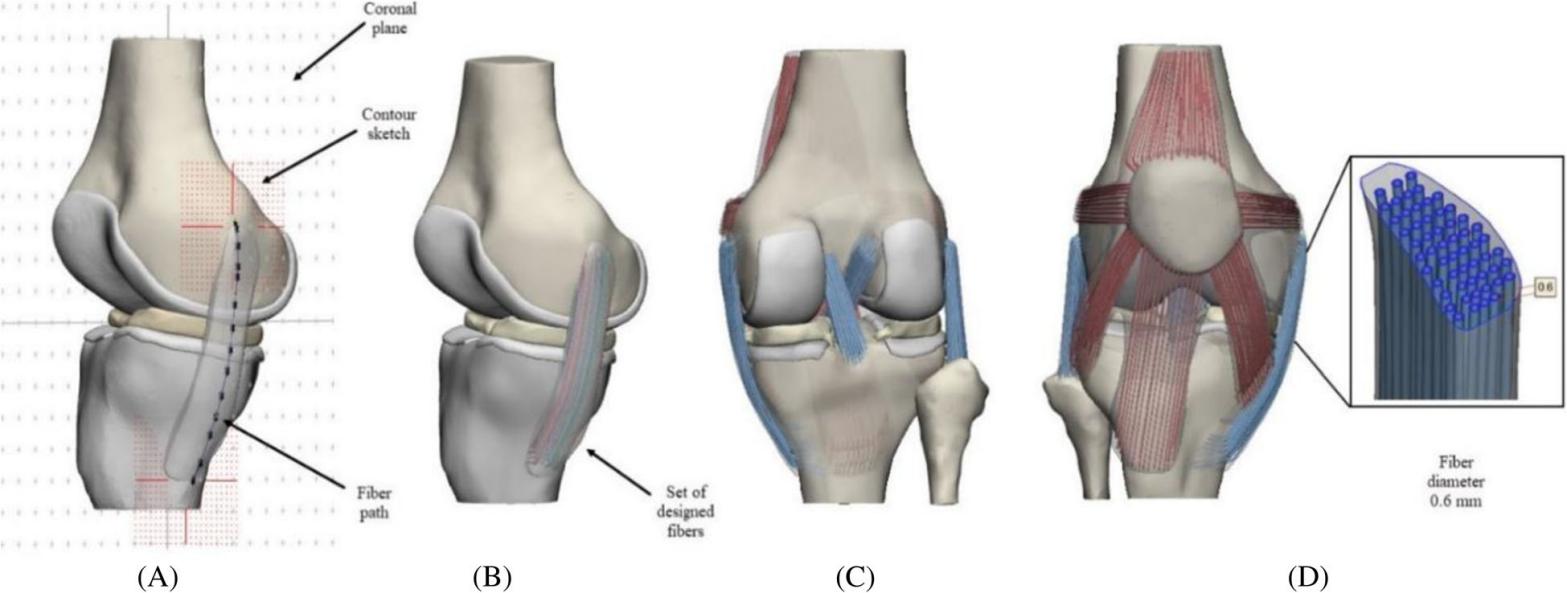
Initial Knee Joint model. **a** Illustration tracing fibers through the medial collateral ligament (MCL), contour and sketches are shown (**b**) collagen fibers set manually created to MCL (**c**) Posterior view of the Knee Joint with all created fibers (**d**) Anterior view of the Knee Joint, cross-section MCL, the fiber diameter is reported

A diameter of 0.6 mm was selected, based on the approximate diameter for the fiber [28]. A tolerance of 0.1 mm was provided considering a possible expansion of the material during the printing process. Successively we used a systematic method to generate contours and paths for each fiber distinct from each other. Fibers were created along with each structure from traced paths using commands Soft curve & Sweet-loft, as shown in Fig. 2. Final matrix fibers structure involved virtual post-processing using Auto-fix, uniform Remesh, Reduce, Smooth & Wrap commands to clean up and correct surface geometry errors, optimize surface mesh and generate a better-refined surface finish for final 3D Printing.

The number of designed fibers for cruciate ligaments, collateral ligaments, PT-QT, medial, and lateral patella-femoral ligaments (MPFL-LPFL), and medial and lateral patellar retinacula (MPR-LPR) were reported in Table 2.

**Table 2:** Number of fibers designed for the Knee Joint Model

Soft tissues	Number of fibers
Anterior cruciate ligament (ACL)	50
Posterior cruciate ligament (PCL)	45
Medial collateral ligament (MCL)	60
Lateral collateral ligament (LCL)	50
Quadriceps tendon (QT)	100
Patellar Tendon (PT) or Patellar Ligament (PL)	45
Medial patella-femoral ligament (MPFL)	30
Lateral patella-femoral ligament (LPFL)	30
Medial patellar retinacula (MPR)	25
Lateral patellar retinacula (LPR)	20

### ACL-R model manufacturing and surgical guide for improving surgical outcomes

**Fig. 3:**
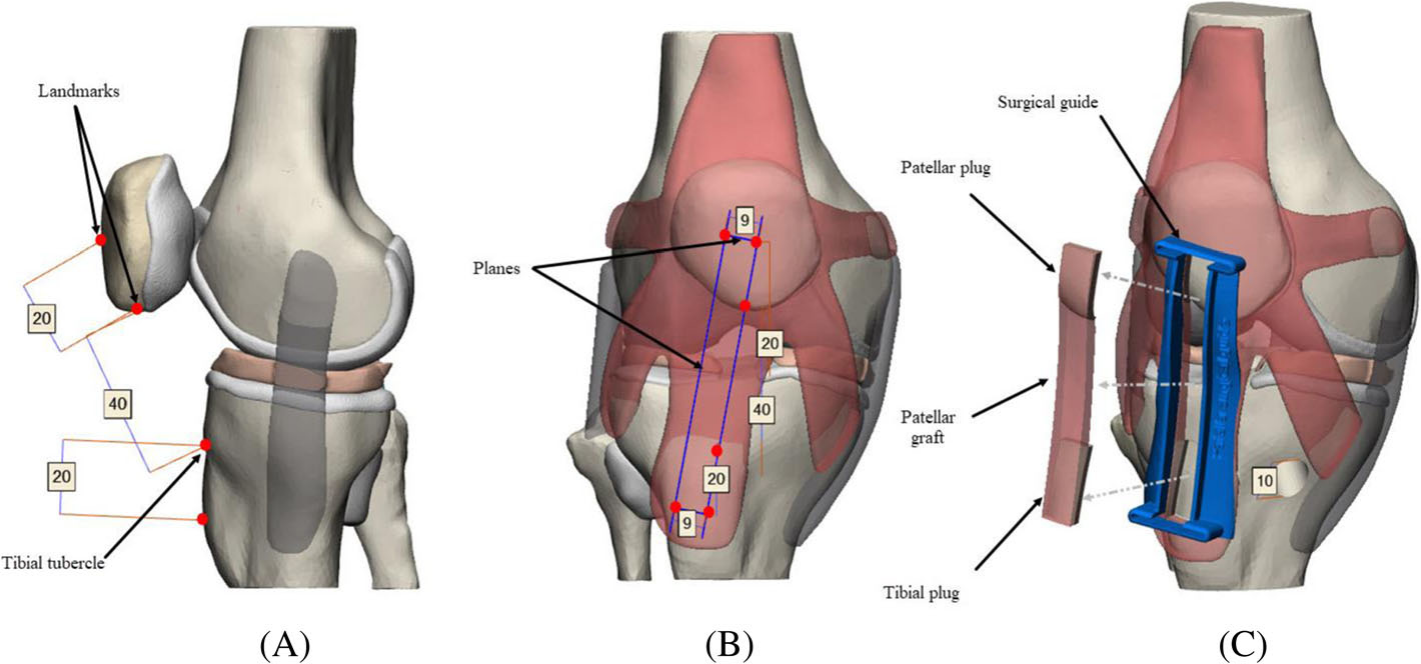
Schematic illustration of the pre-operative measurements and SG positioning. **a** Preliminary measures (mm) and landmarks of BPTB autograft, **b** Simulation of the cut-planes in the graft harvest, **c** Positioned SG, and graft harvest according to the pre-operative guidelines

**Fig. 4:**
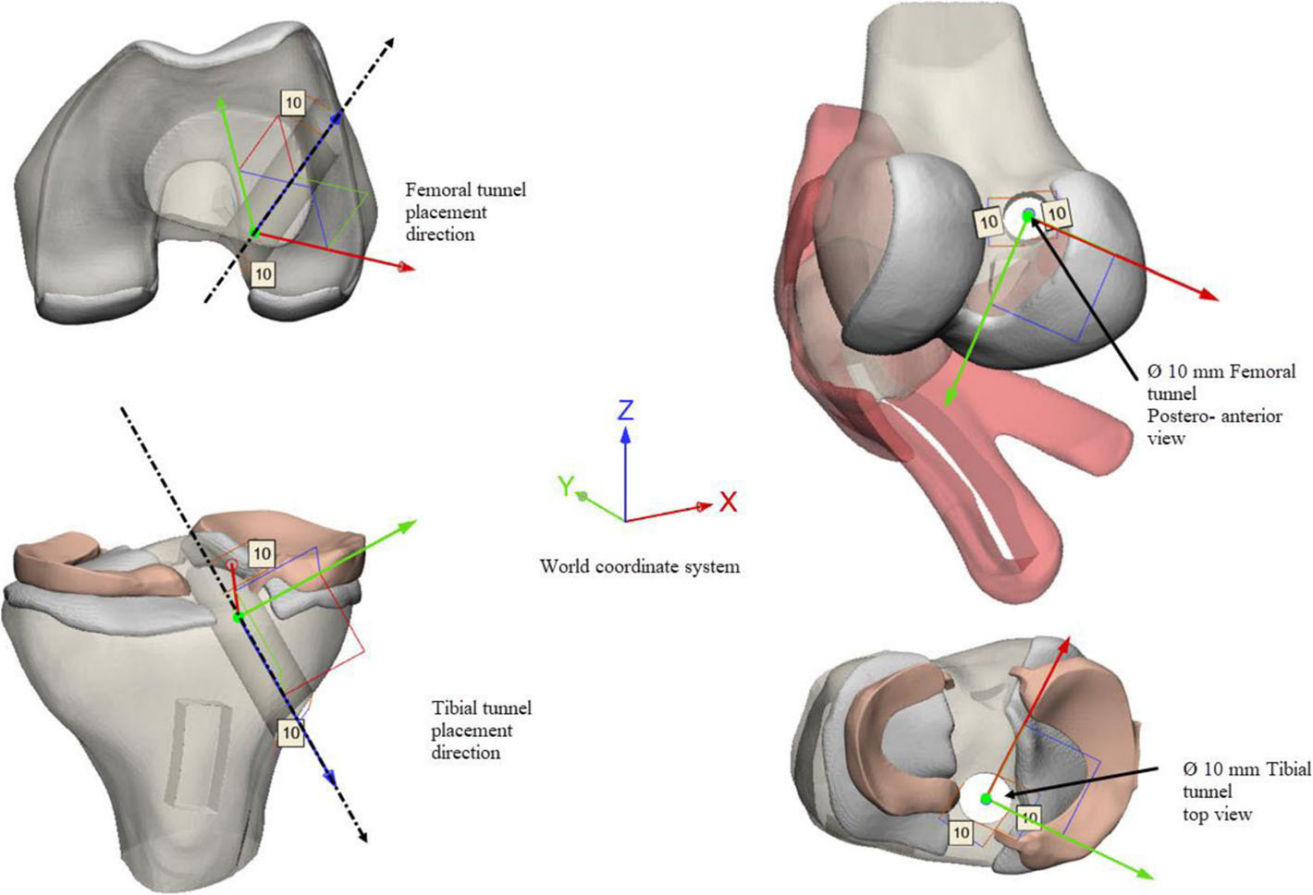
Schematic illustration of the femoral and tibial tunnels architecture; orientations, directions, locations, and dimensions

The statistics of post-operative clinical outcomes represent the definitive proof of success in the treatment of ligament injuries. However, the success of the ACL-R depends on several surgical parameters including graft stiffness, dimensions and pre-tensioning, tunnel placement and orientation, and donor-site morbidity (non-modifiable) [24]. Various specialists give strong importance to the surgical technique, which is associated with adequate medical training and accurate pre-operative planning as well as the graft harvest. We sought the manufacture of a multi-material ACL-R model integrating all key surgical outcomes of the ACL-R computational framework using a BPTB auto-graft and a transtibial technique (TT) with a single-bundle from a predictive model [24]. The approach incorporated orientations, directions, locations, and dimensions of the femoral and tibial tunnels as well as the design of a custom-made SG based on the PT anatomy. The SG aimed to solve the problem associated with auto-graft and tunnel length mismatch. Pre-operative measurements of the BPTB auto-graft length were performed, specifically, the distance, measured from the origin in the lower portion of the patella until its insertion in the tibial tubercle. According to [28], if graft length is greater than or equal to 40 mm, the PT graft is a suitable candidate for replacement. Several authors suggest an average length of 40 mm for graft and 20 mm for each bone plug. The BPTB block must have a rectangular geometry, a width of the graft, and the bone plugs (tibial and patellar) can range between 9 and 11 mm, a width of 9 mm was chosen. The ACL-R model was developed in the Materialise 3-Matic software. The measurements and landmarks were made from the Knee Joint Model with Measure & Landmark commands. Also, we considered for the SG design, the graft harvest using a 0.8mm width oscillating saw blade instead of a traditional scalpel, therefore we provided a 1 mm width for the upper and lower channels. Cut-plans, Boolean tools, marking & extrude commands were involved in the SG design (Fig. 3). Lastly, the graft harvest was performed with Boolean tools & Trim commands following the SG dimensions. Femoral and tibial tunnels of the knee were drilled following the orientations, directions, locations, and dimensions provided in [24] with 10 mm drills (Fig. 4).

### TKA model manufacturing and adjustment of custom-made implants in the initial Knee Joint model

The pre-operative planning by specialized software is an effective tool to decide the type and size of the implant, which predicts the post-operative biomechanical environment and reduces the complications before surgery. The TKA involves three critical implants: femoral, tibial, and articular liners. Each one has an associated criticality however the femoral component is perhaps the most complex of them, usually the design of the rest of the components depends on this implant. The component has a convex shape, which emulates the curvatures of the femoral condyles (located at distal femur) and follows a specific trajectory for each patient (J-curve with a series of distinct radii) fitting correctly with the other implants. Therefore, the correct adaptation of all components provides a high degree of stability throughout the range of motion of the Knee Joint reconstructed and facilitates a close approximation to the native motion of the Knee Joint. In the TKA, the most common cause of failure is aseptic loosening of articular components. When that occurs, all components fail. The wrong relation between implant surfaces is usually the main cause of aseptic loosening. This causes an uneven stress distribution, which leads the component to the failure [18, 29].

The multi-material TKA model was developed in the Materialise 3-Matic software, from the initial Knee Joint model. We chose a custom-made CS implant with an asymmetric tibial bearing fixed design. The model represents the adaptation and suitable relationship of the prosthetic elements following the requirements: (i) the prosthetic components must have the ability to replicate joint motion as closely as possible. (ii) The size of the implants must be custom-made to the actual anatomy of the Knee Joint.

**Fig. 5:**
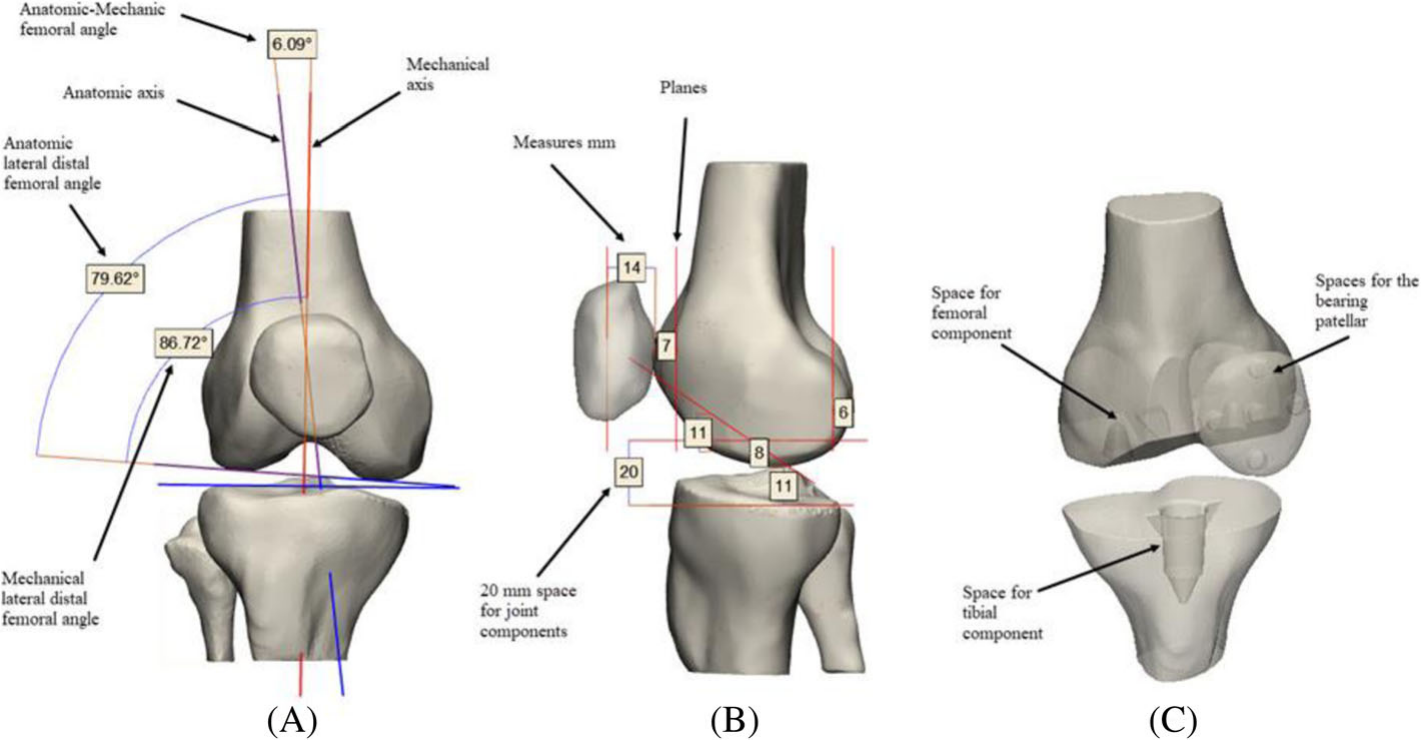
Schematic illustration of the pre-operative measurements and simulated cutting planes. **a** Mechanical and anatomical angles FMAa = 6.09° [5-7°] - FDLAa = 79.62° [79-83°] -FDLMa = 86.72° [85-90°] with their normal angular ranges were reported, **b** Anatomical measurements and simulation of the cutting planes in the bone components, **c** subtraction of the prosthetic components of the bone component according to the planes [30]

First, the anatomical and mechanical axes of the bone components were defined. Pre-operative measurements of the clinical angles were performed using Measure & Landmark commands: anatomic-mechanic femoral angle (FMAa) anatomic lateral distal femoral angle (FDLAa) and mechanical lateral distal femoral angle (FDLMa) [30]. Likewise, measurements of the bone components were performed for the custom-made design of the prosthetic components. Cutting Planes & Trim commands were used to remove bone and cartilage components as follows: 11 mm of the proximal tibia (cross-section), 8 mm of the distal femur (cross-section), 7 mm in the posterior region of the femur (coronal section), 11 mm in the postero-inferior region of the femur (cross-section) and 6 mm in the anterior region of the femur (coronal section), ensuring a space of 20 mm for the replacement of the joint component. The patella was resected 14 mm from the anterior region (coronal section). The pre-operative measurements and simulated cutting planes are illustrated in the Fig. 5.

**Fig. 6:**
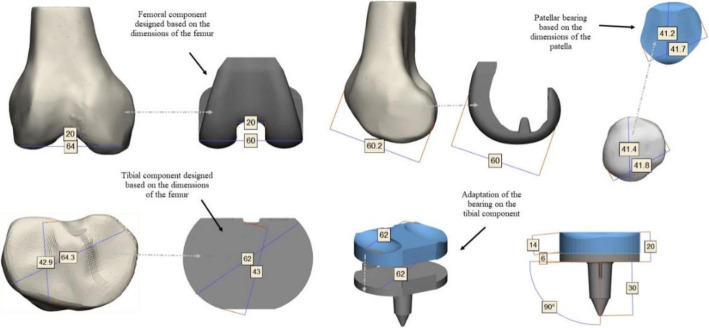
Schematic illustration of the prosthetic components adjustment. Design components were based on the anatomy of the bone components. Patellar and tibial bearings were designed based on the femoral component

**Fig. 7:**
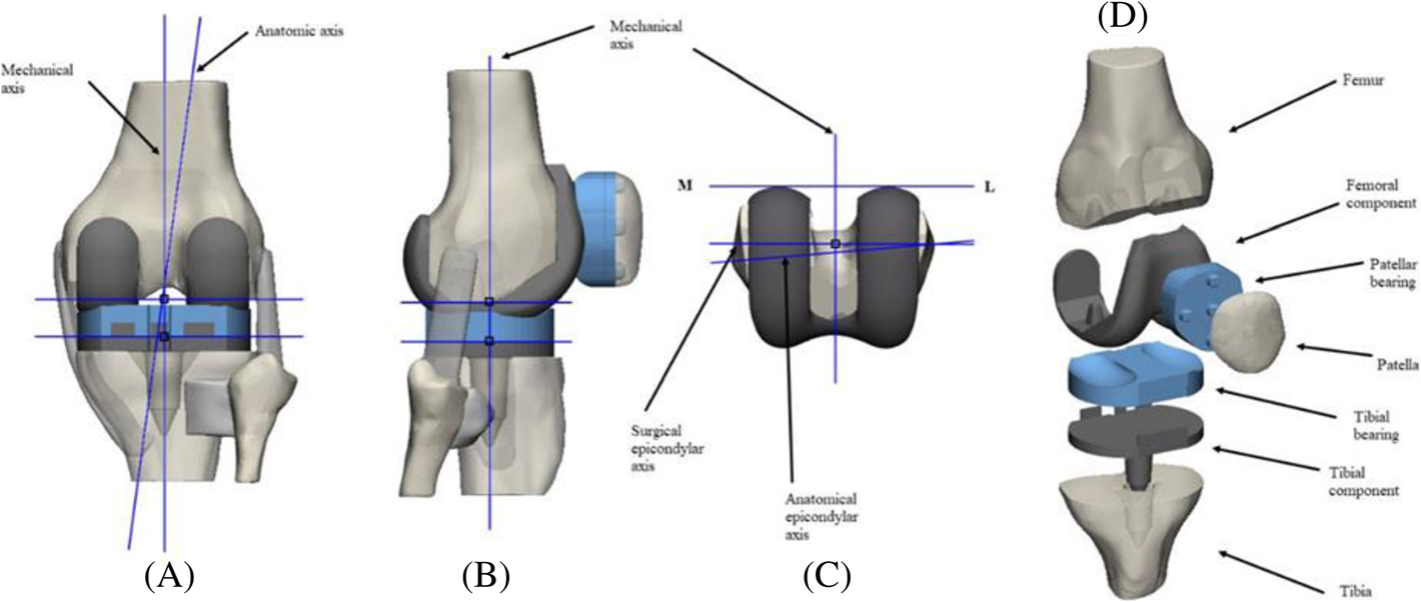
Schematic illustration of the TKA model alignment in a neutral position. **a** Posterior view TKA model, femoral and tibial components are aligned perpendicular to the mechanical axis. **b** Lateral TKA model, the alignment corresponded to 90° concerning the mechanical axis. **c** Transverse view TKA model, the femoral component, anatomic axes-alignment surgical epicondylar axis, and mechanical axis corresponded to 90°. **d** Isometric view assembly of all the components of the TKA model [31]

The femoral and tibial components were exported from Rhinoceros 3D (Robert McNeel & Associates) where they were designed based on bone measurements through standard, Planes & Set View commands (Fig. 6). The implants were adapted to the bone components, the tibial and patellar bearings were designed from them using Boolean & Marking commands. These were adapted to the trajectory and geometry of the femoral and tibial components respectively. In the neutral position, the femoral component was aligned, so that the resection of the distal bone was perpendicular to the mechanical axis of the femur, and the anterior and posterior resections are parallel to each other (Fig. 7).

### Mechanical tensile test and Material selection and matching

To print the proposed Knee Joint anatomical models, a key aspect was to determine appropriate material to mimic real tissue mechanical properties. Therefore, our first approach was to explore printer Stratasys J750 multi-material capabilities. A matching between Shore A hardness scale values of printing available materials and Knee Joint anatomical structures were made. ACL-R model used in the previous study, without considering the designed fiber matrix structure was printed after materials matching (Table 3). The materials: Digital ABS (RGD5130), Agilus30 (FLX935), and Tango (FLX930) were selected for printing bone components and soft tissues respectively. High Mix 27μm layer thickness print mode was set for the model 3D printing. In our model; PT, QT, MPFL, MPFL, MPR, and LPR are a single structure.

**Table 3:** Proposed material matching

Model Structures	Materials selected	Durometer selected (Shore A)
Bones	Digital ABS RGD5130	A80
Ligaments (ACL,PCL,MCL,LCL)	Agilus30 FLX935	A50
Tendons (PT,QT,MPFL,LPFL)	Agilus30 FLX935	A60
Retinacula (MPR, LPR)	Agilus30 FLX935	A60
Menisci	TangoFLX930	A28
Cartilage	TangoFLX930	A28

**Fig. 8:**
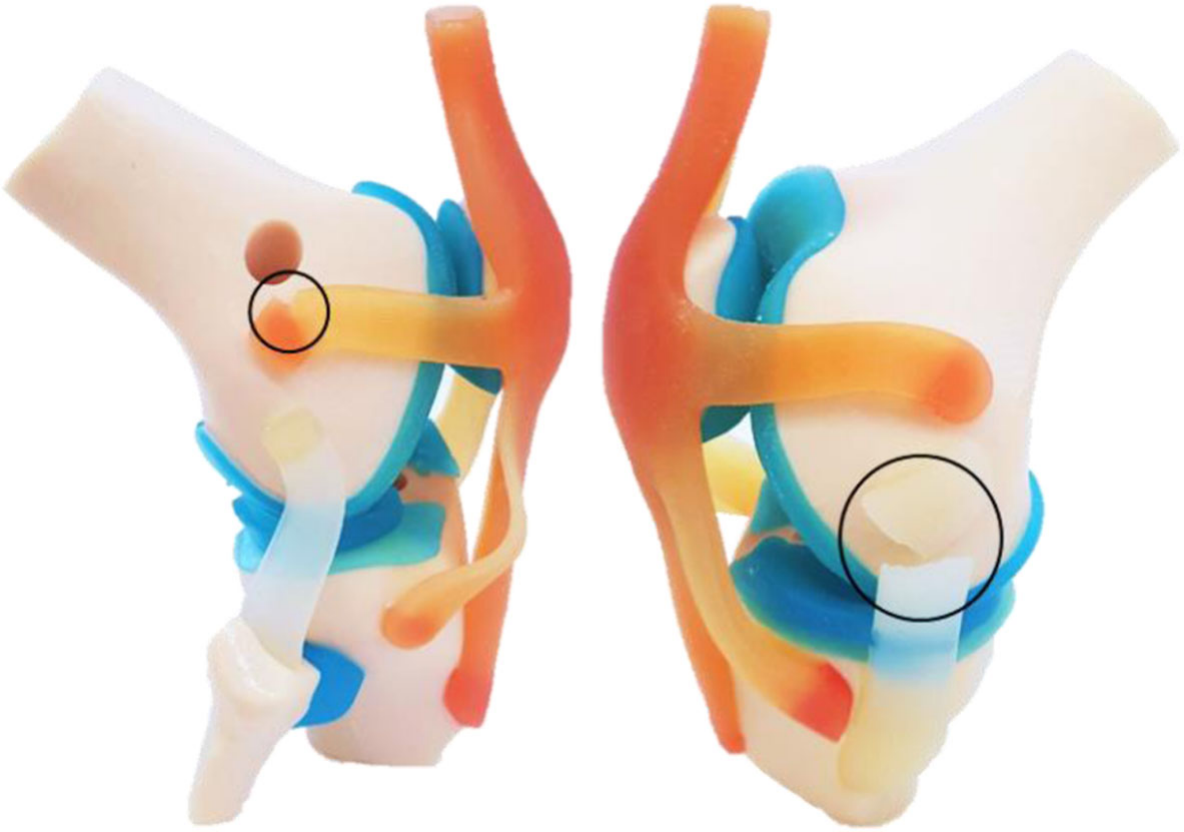
Printing trial scale 1:2. ACL-R model without collagen fibers matrix structure. Knee Joint lateral views in flexion with BPTB graft replacing ACL, rupture of the LPFL, and MCL.

The printing trial result was not satisfactory, ligaments and tendons failed easily after simulating flexo-extension movements (Fig. 8).

**Fig. 9:**
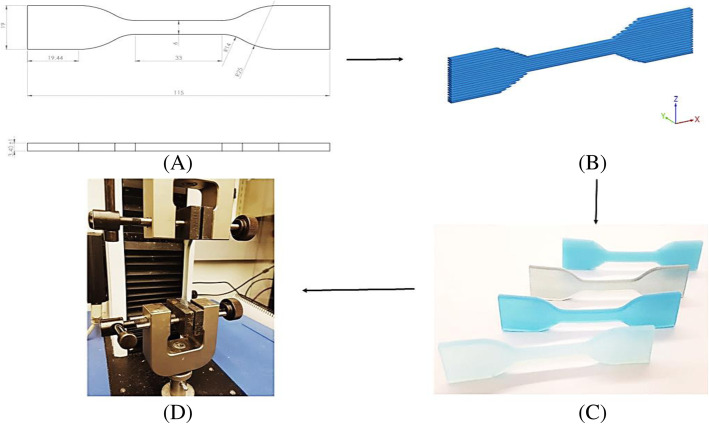
Flowchart application ASTM D412-C standard. **a** Dimensions specimen (mm), **b** Render specimen in SolidWorks software, **c** 3D specimens with different combinations, **d** specimen attached to a materials testing system Instron S3300 uni-axial testing instrument, to apply tensile load

After examining the results of the first printing trial, we included the designed fiber matrix structure inside the soft tissues. Therefore, different combinations of shore-hardness values described in Table 4. between fiber and matrix were proposed. To perform the uniaxial tensile tests, it was necessary to design specimens with fibers inside; we used SolidWorks software following the ASTM D412-C standard specifications (Fig. 9). Three specimens (n=3) were printed perpendicular to the printing tray for each polymer combination. The Mechanical tensile test of the specimens was conducted in Northwestern University Kaiser Lab using an Instron S3300 (Canton, MA) uni-axial testing instrument. Dimensions (thickness, length, width) of each specimen were measured with calibrator; values were set in the software BlueHill, Instrom SA (France, Elancourt). The tensile test was set using a test speed of 10 mm/min. Each specimen was attached between the materials testing system extensometer grips to apply tensile loads. The test was performed until the specimen failed. Data was recorded and exported to Microsoft Excel. The procedure was repeated with all specimens. The Strain-stress curves in the elastic region of each specimen were elaborated in Matlab (MathWorks R2018). From them, mean strain-stress curves, mechanical properties, linear regressions, and Pearson's coefficients R^2^ for each polymer combination were plotted and calculated.

**Table 4:** Different combinations of shore-hardness between fiber and matrix for the tensile test

Material combinations	Fibers Durometer (Shore A)	Matrix Durometer (Shore A)
No. 1	A60	A50
No. 2	A50	A50
No. 3	A50	A40
No. 4	A70	A60

The experimental stress-strain curves of the Knee Joint soft tissues published in literature were analyzed and compiled. Then, the WebPlotDigitizer software [32] was used to reverse engineer images of data visualizations to extract the underlying numerical data. Finally, the data were exported to Microsoft Excel. The comparison of the strain-stress curves was made in Matlab. Likewise, linear regressions in the elastic region (from endpoints of the toe-region to the yield strength point) and Pearson's coefficients were plotted and calculated.

## Results

### Mechanical tensile test

**Fig. 10:**
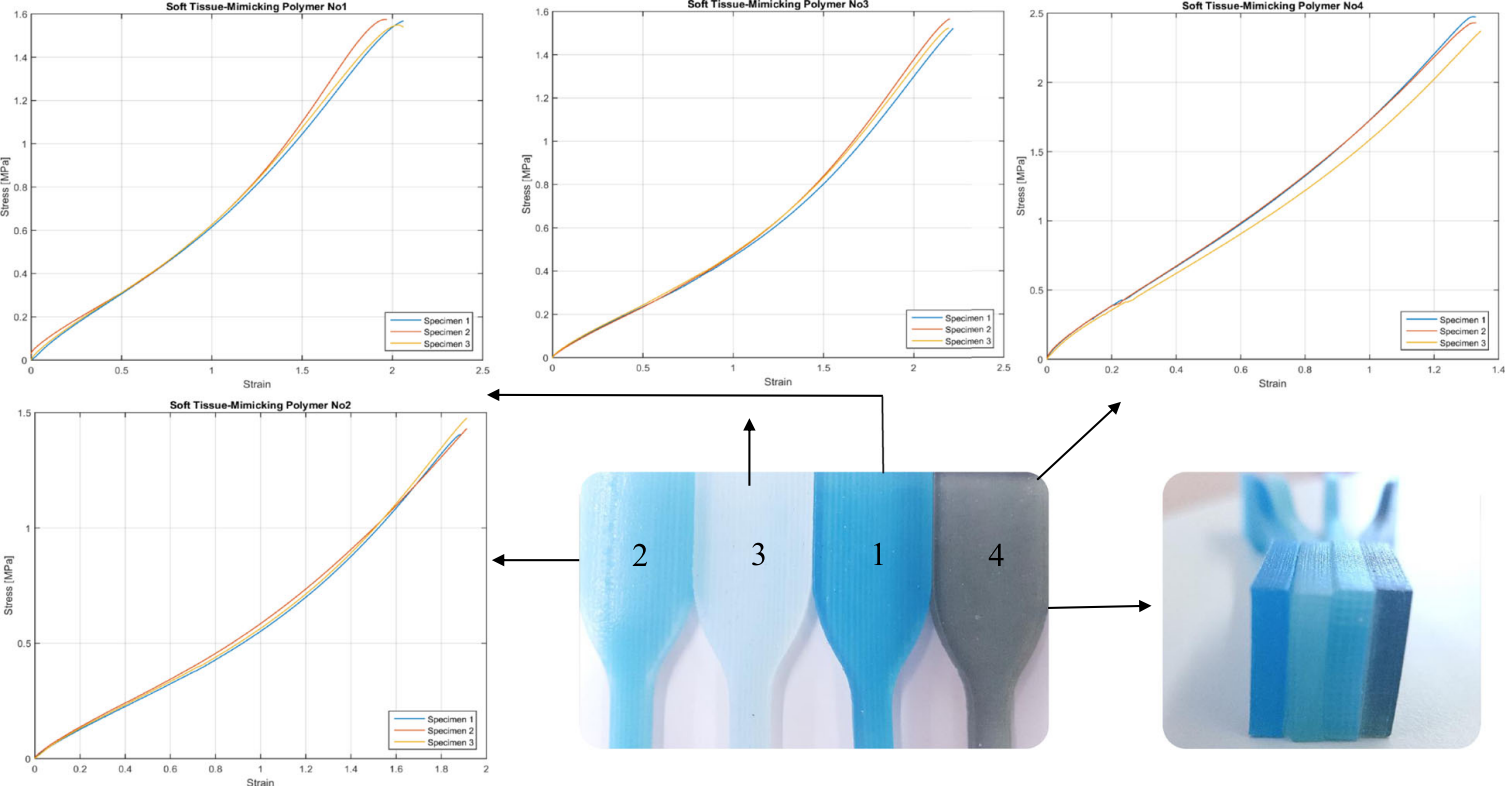
The stress-strain curves of the soft tissue-mimicking polymers (elastic region). Group of specimens No. 1 (Dark Blue), No. 2 (Dark Blue soft), No. 3 (lighter blue), and No. 4 (gray)

To have a better understanding of the mechanical properties and behaviors of soft tissue-mimicking polymers and real soft tissues, the comparison of strain-stress curves, stiffness, yield strengths, and elastic modules were made. Figure 10 presents the stress-strain curves of the proposed combinations obtained from the tensile-test.

**Fig. 11:**
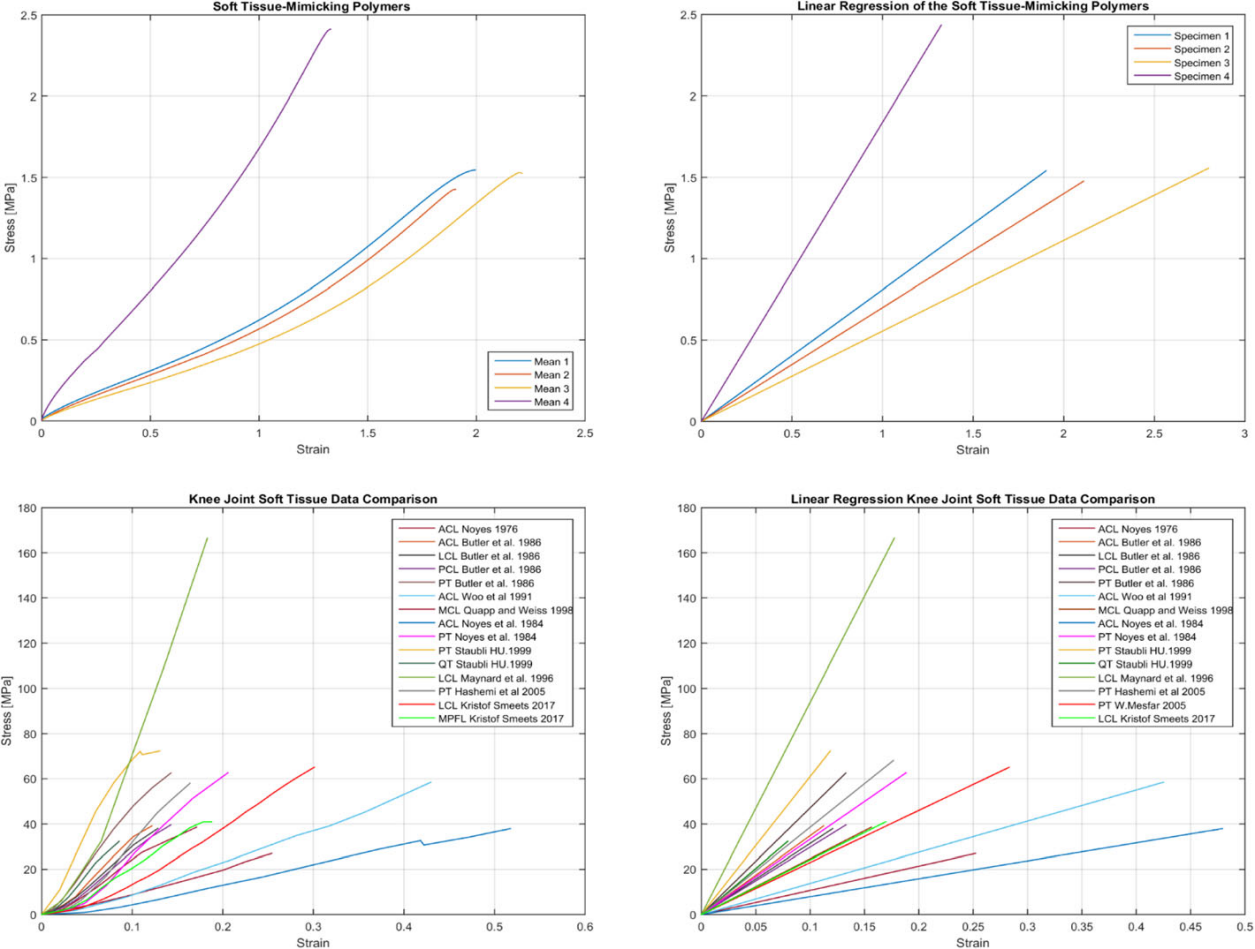
Mean stress-strain curves and linear regressions of the soft tissue-mimicking polymers Knee Joint soft tissues

The stiffness comparison was based on the linear regressions (directly related to the slope of the linear region), reported in Fig. 11. as well as values calculated from dimensions and elastic modulus, and values published in the literature. The linearity of the elastic region was evaluated using Pearson's coefficients, which ranged from 0.980 to 0.990 for Soft tissue-mimicking polymers and 0.878 to 0.989 for real Knee Joint soft tissues. Combinations No. 1 and No. 4 showed higher stiffness of all samples, Knee Joint soft tissues with the higher stiffness were ACL [33] and PT [34]. Tables 5. reports the mean values of elastic modules, yield strengths, standard deviations, and stiffness obtained from the tensile test. Likewise Table 6. reports a data comparison from the published literature for Knee joint soft tissues.

**Table 5:**
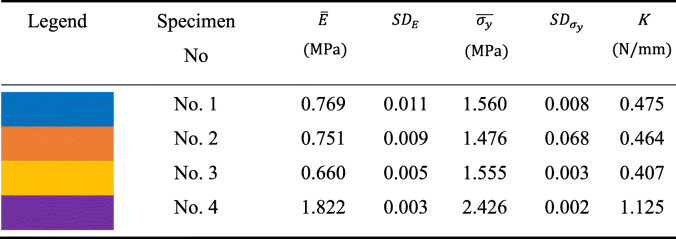
Mean values of Soft tissue-mimicking polymers obtained from the tensile test data, mean elastic modules, yield strengths, standard deviations, and stiffness are reported

**Table 6:**
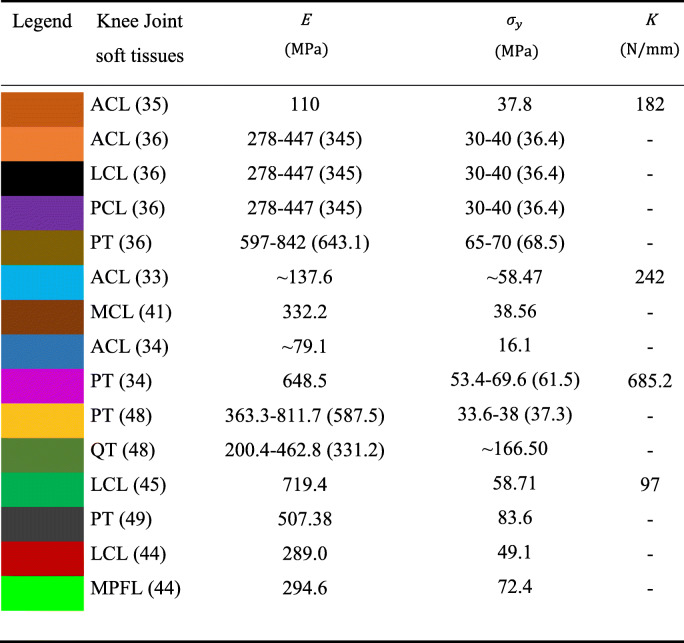
Data comparison of material proprieties from the published literature for Knee Joint soft tissues, the mean value is reported in parentheses

After the tensile test, we identified that No. 1 and No. 4 are the combinations that present the higher values of elastic modulus and stiffness.

After data comparison published in the literature, Mean elastic modulus and stiffness were calculated, including additional studies that do not report strain stress curves (Table 7).

**Table 7:** Elastic modulus and stiffness comparison from different studies

Knee joint soft tissues	Elastic modules reported by different authors (MPa)	$\overline{E}$ (MPa)	Stiffness reported by different authors (MPa)	$\overline{K}$ (N/mm)
ACL	110 [35] , 345 [36], 137,6 [33] 345 [34],	168.05	182 [35], 242 [33], 250 [37] 202 [38]	219
PCL	345 [36], 147.2 [39], 118,7 [38], 109 [40]	179.9	258 [39], 208 [38], 204 [40],	223
MCL	332.2 [41], 463.8 [39], 325.4 [42], 276,9 [43]	349.5	134 [39], 94 [42], 80 [43]	103
LCL	345 [36], 289 [44], 719.4 [45], 183.5 [46]	384.2	33.5 [46], 97 [45], 60 [47]	63.5
PT	643.1 [36], 648.5 [34], 587.5 [48], 507.38 [49]	596.62	685 [34], 555.5 [50]	620.25
QT	331.2 [48], 462.8 [51]	397	-	-

The Knee Joint soft tissues with higher elastic modulus were PT, MCL, LCL, and QT. The cruciate ligaments showed higher stiffness.

### 3D Printing of the Knee Joint anatomical models

Three Knee Joint models were printed: (i) ACL-R model used in the previous study. (ii) ACL-R model, incorporating orientations, directions, locations, and dimensions of the tunnels, as well as a custom-made SG. (iii) Total knee arthroplasty (TKA) model, including custom-made implants. Agilus30 (FLX930) printing material with the combinations No. 1 (A60 fibers and A50 matrix) – No. 4 (A70 fibers and A60 matrix) were chosen to print Knee Joint soft tissues according to the matching reported in Table 8. Tango FLX930 (A28) - FLX950 (A75) printing materials were chosen to print menisci, cartilage surfaces, and articular liners respectively. Finally, Digital ABS RGD5130 (A95) was chosen to print bone components, SG, and implants.

**Table 8:** reports mean values of elastic modulus, stiffness, and proposed matching with the combinations for the PolyJet 3D printing

Knee Joint soft tissues	$\overline{E}$ (MPa)	$\overline{K}$ (N/mm)	Selected combination	$\overline{K}$ (N/mm)	$\overline{E}$ (MPa)
PT	596.62	620.25	No. 4	1.125	1.82
QT	397	-	No. 4	1.125	1.82
MCL	349.5	103	No. 4	1.125	1.82
MPFL, LPFL, MPR, LPR	-	-	No. 4	1.125	1.82
LCL	384.2	63.5	No. 1	0.475	0.76
PCL	179.9	223	No. 1	0.475	0.76
ACL	168.05	219	No. 1	0.475	0.76

**Fig. 12:**
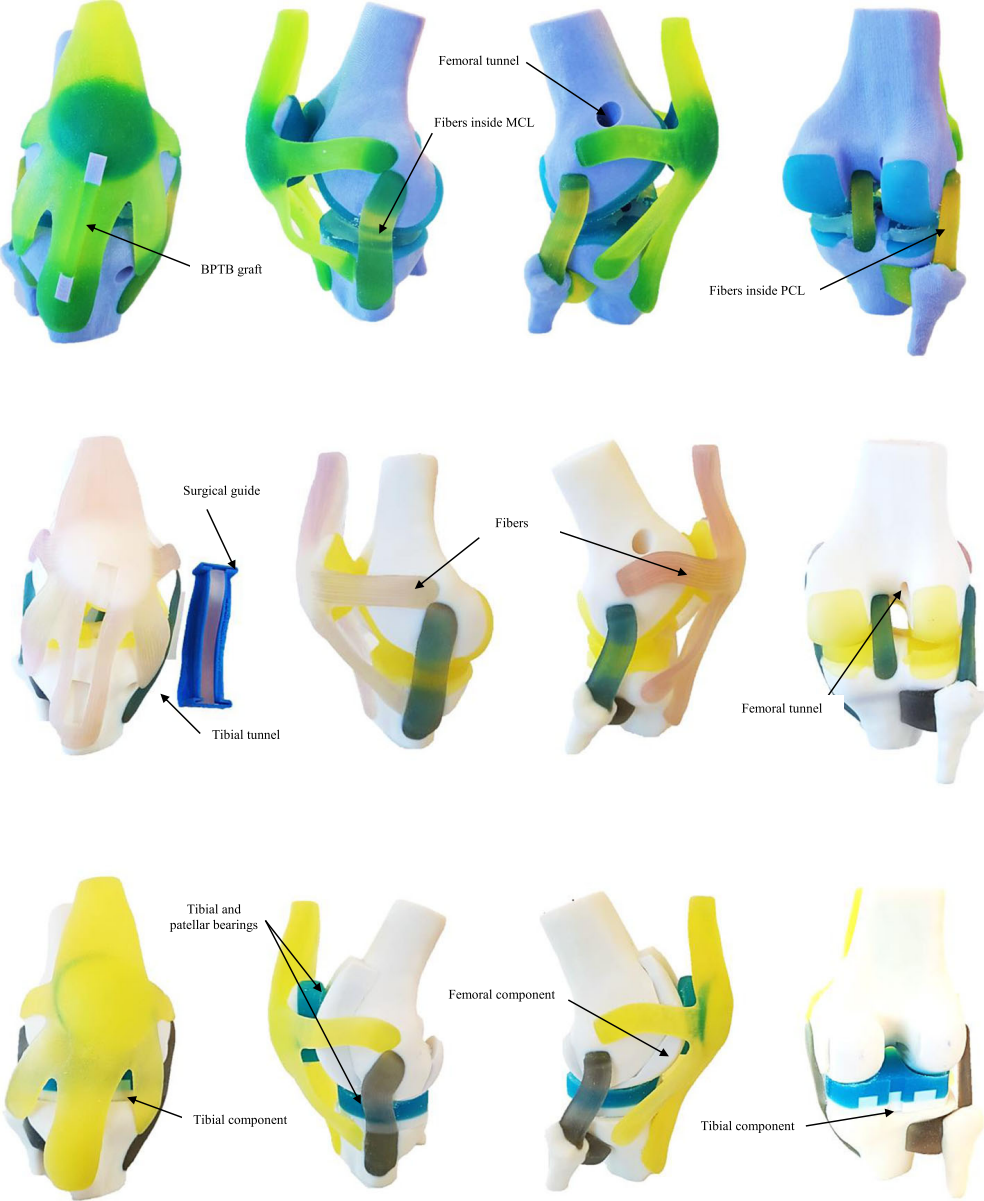
Illustration of 3D printing Knee Joint anatomical models, Anterior-Lateral- Posterior views of (i) ACL-R* previous study, (ii) ACL-R and SG, and (iii) TKA model. Models show fibers matrix structure inside soft tissue structures

The PolyJet 3D printing enabled the combination of hard and elastic materials in a single project. After the completion of printing, wax-like PolyJet support material SUP706 [23] was removed using a pressure water gun, the whole process took about 5 minutes. Finally, anatomical models were evaluated manually simulating 50 flexo-extension cycles using a three-in-one multi-purpose oil (ACL-R model was tested without BPTB positioned). The 3D printing Knee joint models did not present rupture or wear in their connective structures or at the insertion points. The custom-made SG matches the anatomy of the Knee Joint in the ACL-R model as it was expected (Fig. 12). Likewise, the BPTB measurements match with the SG dimensions. The custom-made implants of the TKA model accomplished its requirements established preliminary planning but the range of motion was more limited.

## Discussion

The current study aimed to fabricate multi-color and multi-material realistic Knee Joint anatomical models with unique features. In particular, the design of a fibers matrix structure that mimics the soft tissue anatomy.

From the experimental data of the uniaxial tensile test, characteristic graphs of each group of combinations were obtained (Fig. 10). The behavior of the strain-stress curves reported showed a mechanical pattern similar and approximate linear behavior to the graphs obtained from the published literature (Fig. 11). Mean curves, linear regressions, and mechanical properties (Table 5) were obtained from them. Combinations No. 1 and No. 4 with a fiber–matrix shore hardness 60-50 and 60-70, elastic modulus of 0.76- 1.82, and stiffness of 0.475- 1.125 respectively showed the higher and appropriate values of all combinations, the following was verified through the slope of the linear regression graphs (purple and yellow straight lines). Additionally, we concluded that the test results are reliable and repeatable since the standard deviations found vary between 0.003 to 0.011 and 0.002 to 0.008 for elastic modules and stiffness of the combinations proposed for Polyjet 3D printing of Knee Joint models. Likewise, the values of the Pearson’s coefficients that varied from 0.980 to 0.990 showed a linear correlation of the data in the elastic region of the materials, which means that data adjustment was a good approximation of the linear region, representative of the elastic behavior of ligaments and tendons.

We analyzed the data reported in the literature about the structures present in the Knee Joint, we found several outlier values reported by the different authors (Table 6) [33–36, 41, 44, 45, 48, 49]. Therefore, we calculated a mean elastic modulus and stiffness from additional studies where the values were reported but not the graphs (Table 7). We found that The Knee Joint soft tissues with higher elastic modulus were PT, MCL, LCL, QT, and cruciate ligaments had higher stiffness. As the data have great variability due to the type of specimen, experimental setup, test conditions, and type of donor, we analyzed the linear regressions (Fig. 11) searching soft tissues with greater slope. We conclude that Knee Joint soft tissues with higher elastic modulus and stiffness were PT (yellow), MCL (brown), LCL (light green), and QT (dark green). We assigned to these structures the combination No. 4 while the others with less modulus and stiffness we assigned the combination No. 1.

From a functional point of view, we compared the initial ACL-R model (without hierarchical structure) and ACL-R proposed model. The new ACL-R model was more realistic than the previous, integrating appropriate graft dimensions reported in literature an SG. In general, the proposed Knee Joint models withstood repetitive flexo-extension cycles without tear or ruptures (Fig. 12).

**Fig. 13:**
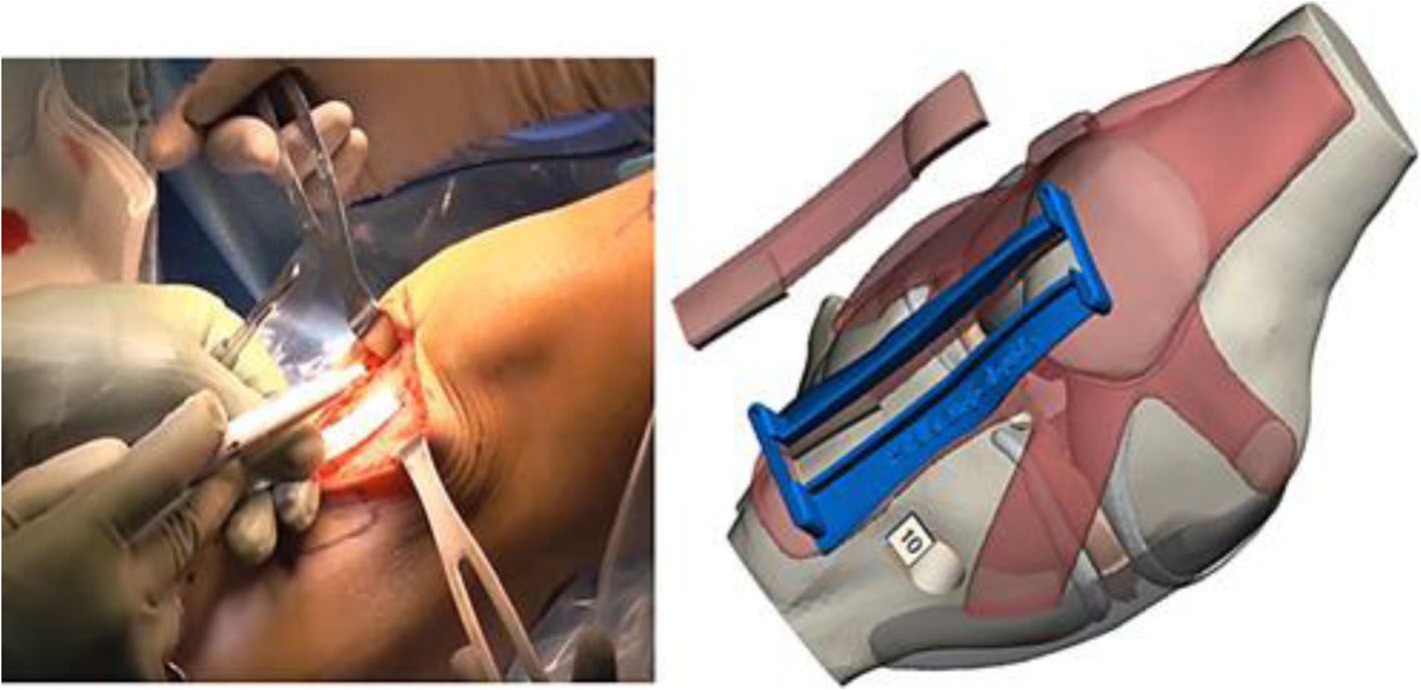
Traditional approach for BPTB autograft harvest using a ruler to measure graft dimension and proposed approach using SG

The custom-made ACL printed SG matches with the anatomy, this would enable the surgeon to solve the problem related to graft tunnel length mismatch and uncertainty in the graft harvest, ensuring dimensions established in the pre-operative plan. The main limitation of this approach was surgical validation, it is expected that the SG may be used in the application of real cases of ACL-R. Figure 13. Shows the comparison between the traditional approach and the proposed solution.

Finally, in our study, the purpose of the TKA model was to fulfill the functional requirements established in preoperative planning. There were two main limitations; the designed implants considered an extreme case of OA in which the cruciate ligaments are sacrificed, which is not recommended from the medical criteria. Besides, the approach was based on a Knee Joint under normal conditions and it does not represent a real OA condition, therefore, the implants proposed are not a real application for TKA, these are useful only for educational proposes. Several critical factors determine the success or failure of TKA that this study does not consider.

## Conclusion

The proposed anatomical models offer a diverse range of applications. These may be considered as an alternative to replacing cadaver specimens for medical training, pre-operative planning, research and education purposes, and predictive models validation. We highlighted that mechanical patterns and stiffness obtained for the soft tissue-mimicking polymers are comparable qualitatively, despite different elastic modulus and linear stiffness values. The results showed that the proposed soft tissue anatomy-mimicking materials are strong enough to withstand the stretching during the flexo-extension. The methodology reported for the design of the fiber-matrix structure might be considered as a start to develop new patterns and typologies that may mimic soft tissues.

## Data Availability

The STL files of anatomical models and experimental data used to support the findings of this study are available from the corresponding author on reasonable request.

## Abbreviations

CAD: Computer-aided design
CAE: Computer-aided engineering
TKA: Total knee arthroplasty
PSI: Patient-specific instrumentation
ACL: Anterior cruciate ligament
ACL-R: Anterior cruciate ligament reconstruction
SG: Surgical guide
OA: Osteoarthritis
BPTB: Bone-patellar tendon-bone
CS: Cruciate sacrificing
UV: Ultraviolet
LT: Layer thickness
ABS: Acrylonitrile butadiene styrene
FDM: Fused Deposition Modeling
STL: Standard triangle language
PF: Patello-femoral
TF: Tibio-femoral
PT: Patella tendon
QT: Quadricep tendon
GRS: Global reference system
PCL: Posterior cruciate ligament
MCL: Medial collateral ligament
LCL: Lateral collateral ligament
MPFL: Medial patella-femoral ligament
LPFL: Lateral patella-femoral ligament
MPR: Medial patellar retinacula
LPR: Lateral patellar retinacula
TT: Transtibial technique
FMAa: Anatomic-mechanic femoral angle
FDLAa: Anatomic lateral distal femoral angle
FDLMa: Mechanical lateral distal femoral angle
